# The Role of *S. cerevisiae* Sub1/PC4 in Transcription Elongation Depends on the C-Terminal Region and Is Independent of the ssDNA Binding Domain

**DOI:** 10.3390/cells11203320

**Published:** 2022-10-21

**Authors:** Alejandro Collin, Araceli González-Jiménez, María del Carmen González-Jiménez, Manuel J. Alfonso, Olga Calvo

**Affiliations:** 1 Cátedra de Bioquímica y Biología Molecular, Facultad de Ciencias Médicas-INICSA, CONICET-Universidad Nacional de Córdoba, Haya de la Torre s/n, Pabellón Argentina, 2º piso. Ciudad Universitaria, Cordoba CP5000, Argentina; 2 Instituto de Biología Funcional y Genómica (IBFG), CSIC-USAL, C/ Zacarías González, nº2, 37007 Salamanca, Spain

**Keywords:** Sub1, PC4, DNA binding proteins, RNA polymerase II, transcription elongation, *Saccharomyces cerevisiae*

## Abstract

*Saccharomyces cerevisiae* Sub1 (ScSub1) has been defined as a transcriptional stimulatory protein due to its homology to the ssDNA binding domain (ssDBD) of human PC4 (hPC4). Recently, PC4/Sub1 orthologues have been elucidated in eukaryotes, prokaryotes, and bacteriophages with functions related to DNA metabolism. Additionally, ScSub1 contains a unique carboxyl–terminal region (CT) of unknown function up to date. Specifically, it has been shown that Sub1 is required for transcription activation, as well as other processes, throughout the transcription cycle. Despite the progress that has been made in understanding the mechanism underlying Sub1′s functions, some questions remain unanswered. As a case in point: whether Sub1’s roles in initiation and elongation are differentially predicated on distinct regions of the protein or how Sub1′s functions are regulated. Here, we uncover some residues that are key for DNA–ScSub1 interaction in vivo, localized in the ssDBD, and required for Sub1 recruitment to promoters. Furthermore, using an array of genetic and molecular techniques, we demonstrate that the CT region is required for transcription elongation by RNA polymerase II (RNAPII). Altogether, our data indicate that Sub1 plays a dual role during transcription—in initiation through the ssDBD and in elongation through the CT region.

## 1. Introduction

RNAPII transcription progresses through highly regulated steps assisted by a considerable number of auxiliary proteins: general transcription factors, elongation and termination factors, and a diversity of complexes working as activators, repressors, co-activators, and co-repressors [1]. However, partly due to the lack of knowledge of their structure and properties, the functional interactions carried out by some of these proteins are still not well understood. These proteins include the transcriptional co-activator Sub1 in *S. cerevisiae*, which was initially explicated as a transcriptional stimulatory protein, owing to its homology to hPC4, its ability to activate transcription [2,3,4], as well as the physical and genetic interactions with the general transcription factor TFIIB [5,6].

The homology of ScSub1 and PC4 resides in a ssDBD, which has also been described in other organisms [7], such as in the fission yeast *Schizosaccharomyces pombe* [8] and the rice blast fungus *Magnaporthe oryzae* [9]. In addition, PC4/Sub1 orthologs have been identified in prokaryotes such as in *Lactococcus lactis* [10], *Burkholderia pseudomallei* [11], and even in the bacteriophage T5 [12]. It is also pertinent to note that structures of the DNA binding domains of PC4 [13,14,15], MoSub1 [7,9,16], *L. lactis,* and bacteriophage T5 have been elucidated. In the case of PC4, it forms homodimers interacting with a 5-nucleotide region of two opposing DNA strands [13,14,17]. Responsible for basal and activated transcription, the PC4 ssDBD has also been involved in transcription repression when PC4 levels are increased [14]. PC4/Sub1 also functions in RNAPIII transcription termination and reinitiation [18], as well as DNA metabolism, e.g., replication and DNA repair and, therefore, genome stability (reviewed in [19], and references therein). Each of these functions is directly related to the ssDBD. Exclusively, budding yeast Sub1 contains an extra-long carboxyl–terminal region (CT) of unknown function not found in other organisms. Due to this additional region, ScSub1 is suggested to have functional differences. 

ScSub1 has been shown to participate in many processes, during mRNA biogenesis, all along the transcription cycle [19,20]. For instance, during initiation, it participates in the activation of osmo-response genes upon osmotic shock [21], the regulation of *IMD2* expression [22], and the selection of the transcription start site [23]. In fact, Sub1 has been described as a preinitiation complex (PIC) component [24]. Additionally, Sub1 modulates RNAPII phosphorylation during the whole transcription cycle [25], impacts transcription elongation rate and splicing [26], and by interacting with the 3′-end processing factor, Rna15, it influences transcription termination. Sub1–Rna15 interaction is evolutionarily conserved in mammals through PC4 association with the polyadenylation factor Cstf64 [27,28]. More recently, it has been shown that Sub1 interacts with the RNAPII stalk domain, consisting of Rpb4 and Rpb7 subunits, which could explain Sub1′s roles in transcription [29]. Whether only the ssDBD region of Sub1 protein is the key to participating in all these processes or, instead, it requires its unusual CT region is still an open question. In this work, we have achieved a functional analysis of the Sub1 ssDBD, identifying essential residues for Sub1 binding to the DNA and confirming its evolutionary relevance. In addition, we provide data demonstrating that in the CT region of Sub1 resides the ability to function as a transcription elongation factor. Thus, our data indicate that Sub1 plays a dual role in transcription, being an initiation factor through its ssDBD and a transcription elongation factor through its CT region.

## 2. Materials and Methods

### 2.1. Yeast Strains, Media and Generation of Strains

The strains used are listed in supplementary Table S1. Strain construction and other genetic manipulations were performed following standard procedures [30].

DNA binding mutations were created by site-directed mutagenesis of a wild-type copy of *SUB1*, cloned into a centromeric plasmid under its own promoter, and with six copies of the HA epitope at its carboxyl–terminal for its detection. Mutant and wild-type strains were generated by transforming a *sub1*∆ strain lacking *SUB1,* with plasmids containing mutated or *wt* copies of *SUB1*, respectively. Deletion of the Sub1 carboxyl–terminal region was performed by homolog recombination directed to the chromosomal copy of *SUB1* and replaced with 6xHA. For some experiments, a *sub1*∆ strain, transformed with a centromeric plasmid containing *sub1*∆*CT*, was also used. 

### 2.2. Co-Immunoprecipitation and Western Blot Analysis

Cells containing HA-tagged Sub1 were grown at 28 °C in 200 mL of a rich medium or an SC medium to an OD600 of 1.0, harvested, washed with water, and followed by suspension in 1.5 mL of lysis buffer (20 mM HEPES, pH 7.6, 200 mM potassium acetate, 1 mM EDTA pH 8.0, glycerol 10%) containing protease and phosphatase inhibitors. The cell suspension was flash frozen in liquid nitrogen and then ground, in Spex Freezer Mill 6775, to a fine powder. Afterward, the cell lysate was thawed slowly on ice, transferred to pre-chilled tubes, and centrifuged at 13.200 rpm for 20 min. The supernatant was collected, and total protein concentration was estimated by measuring absorbance at 280 nm in a nanodrop. Exactly 1 μL of anti-HA was incubated with 25 μL of magnetic beads (Dynabeads™ M-280 Sheep Anti-Mouse IgG, Invitrogen-Thermo Fisher Scientific, Waltham, MA, USA) for 1 h at 4 °C in PBS/0.1% BSA. After washing, the antibody coupled to beads was incubated, with whole cell extracts containing the corresponding amount of protein, for 2 h at 4 °C. The IPs were extensively washed with lysis buffer, and beads were suspended in an SDS-PAGE sample buffer. Thereafter, they were incubated at 65 °C for 5 min, and supernatants were loaded onto an SDS-PAGE gel.

Western blot analysis was performed using appropriate antibodies in each case. Anti-phosphoglycerate kinase (Pgk1, 459250, Invitrogen), anti-HA (12CA5, Roche, Basilea, Switzerland), and anti-Rpb1 (8WG16, Covance, Princeton, New Jersey, USA) were acquired from the indicated vendors. ECL reagents were used for detection. The signal was acquired on film and/or with a ChemiDoc XRS (Bio-Rad) system and, when necessary, quantified with the Quantity One software (Bio-Rad). The data plotted correspond to mean values from at least three different experiments, and the error bars represent standard deviations.

### 2.3. Chromatin Immunoprecipitation (ChIP)

Chromatin purification, immunoprecipitation (IP), quantitative real-time PCR (qPCR) amplification, and data analysis were performed as previously described [26,29,31]. Briefly, PCR of purified chromatin, following immunoprecipitation, was performed by quantitative real-time PCR with the CFX96 Detection System (Bio-Rad Laboratories, Inc. Hercules, California, USA), using TB Green^®^ Premix Ex Taq™ (Tli RNaseH Plus) from Takara Bio Inc (Göteborg, Sweden), following the manufacturer’s instructions. The four serial 10-fold dilutions of genomic DNA were amplified, using the same reaction mixture as the samples, to construct the standard curves. Real-time PCR reactions were performed in triplicate using at least three independent ChIPs. Quantitative analysis was carried out using the CFX96 Manager software (version 3.1, Bio-Rad Laboratories, Inc. Hercules, California, USA). The values obtained for the IP PCR products were compared to those of the total input, and the ratio of the values from each PCR product, from transcribed genes to a non-transcribed region of chromosome VII or to chromatin samples incubated with beads but no antibody, was calculated. Numbers on the y-axis of graphs are detailed in the corresponding figure legend.

### 2.4. Transcriptional Run-On (TRO)

TRO assay was performed, essentially, as described in [26,27,32]. Next, 25 milliliters of the corresponding strains, grown at 28 °C up to an OD600 of 0.3–4, were induced with 2% galactose, permeabilized, and nascent RNA-labeled with [α-32P]UTP for 5 min at 30 °C. After partial hydrolysis, RNA was hybridized directly to filter immobilized *FMPL27* probes. Hybridization signals were quantitated by PhosphorImager analysis (Personal Molecular Imager; Bio-Rad Laboratories, Inc. Hercules, California, USA). The 18S rRNA signal was used for normalization, and then, the results were compared relative to the ATG probe, which was fixed at 1. Each experiment was performed at least three times.

### 2.5. RNA Isolation and RT-PCR

Total RNA was extracted as described by [33], and RT-PCR was performed using the iScript RT reagent Kit (Bio-Rad Laboratories, Inc. Hercules, California, USA) following the manufacturer’s instructions. PCR or qPCR reactions were performed in triplicate with at least three independent cDNA samples. PCR products were run on an ethidium bromide-stained gel. 

### 2.6. GLAM Assay

The GLAM assay was carried out as in [34], with cells grown at 28 °C to the mid-log phase in a selective synthetic medium (SC) containing 2% galactose and lacking uracil. Acidic phosphatase activity was measured at least twice in cells grown in three to four independently induced cultures for each strain. The mean values and standard deviations are represented in the corresponding figures.

### 2.7. 6 -Azauracil Induction (6AU)

The cells were grown at 28 °C in SC media, without uracil, to an OD600 of 0.5, split into two cultures, and 6AU was added to one of them to a final concentration of 100 μg/mL. Both cultures were incubated for 1 h, collected, washed, and resuspended in the appropriate buffer to isolate RNA. RT-qPCR was performed as described above. 

### 2.8. Statistical Analysis

All experiments were performed at least in triplicate (n ≥ 3). The data were previously normalized with the square root and, then, scaled by the Pareto method. Data were processed using the statistical package Statgraphics Centurion XVI.II, from STSC, Inc. (Rockville, MD, USA). The tests applied were the one-way analysis of variance (ANOVA) and the Fisher’s test to establish Homogeneous Groups at a significance level *p* ≤ 0.05.

### 2.9. Sequence Alignment, Prediction and Modelling of Sc and Sp Sub1 Structures 

Multiple sequence alignment was generated using CLUSTALW and represented with Jalview program, version 2.11.2.5 (University of Dundee, _Scotland, UK) 

Crystal structure of the ssDNA-binding domain homodimers from hPC4 and MoSub1 structures were obtained with the following ID codes: PDB 7E4W [35] and PDB 4AGH [9], respectively. In the case of the yeast orthologues, ScSub1 and SpSub1 ssDBD dimer structures (40–105 and 40–94) were predicted using the Alphafold2 program [36] and superimposed to hPC4 and MoSub1 structures using the UCSF Chimera program, version 1.16 (University of California, San Francisco, USA) [37]. Hhpred interactive server, for protein homology detection and structure prediction, was used to model ScSub1 structure bound to the ssDNA (https://toolkit.tuebingen.mpg.de/tools/hhpred, accessed on 16 March 2019). 

## 3. Results

### 3.1. Sub1 ssDNA Binding Domain Shares Homology and Binding Capacity with Human PC4

**Figure 1:**
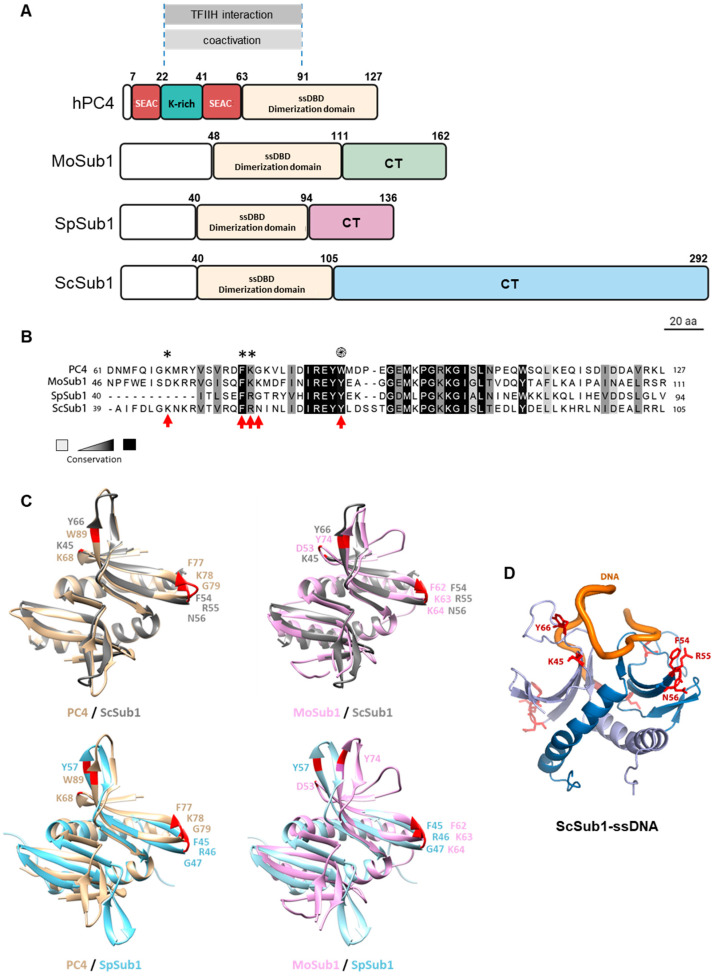
The DNA binding domain of *Saccharomyces cerevisiae* Sub1 is evolutionarily conserved. (**A**) A schematic representation of the primary structures of human PC4 (hPC4) and Sub1 orthologues from *Saccharomyces cerevisiae* (ScSub1), *Schizosaccharomyces pombe* (SpSub1), and *Magnaporthe oryzae* (MoSub1) showing the size and location of their structural and functional domains; hPC4 contains a lysine-rich (K-rich) region bordered by two serine and acidic residue-rich (SEAC) regions. The region for TFIIH interaction and coactivation is indicated. (**B**) Multiple sequence alignment of the ssDBD/dimerization region of hPC4/Sub1 orthologs showing the highly conserved residues; a white/black scale is shown to indicate residue conservation; thus, highly conserved residues are shown within a black background. In the case of ScSub1 ssDBD, 49% of the residues are identical and 26.9% are similar to hPC4 ssDBD amino acids. Additionally, (*,**) indicate the residues, the mutation of which reduces the binding of PC4 to ssDNA, and (^֍^) indicates W89 residues, whose mutation causes loss of DNA binding [14]. The conserved residues that have been mutated in ScSub1 in our study are indicated by red arrows. (**C**) ScSub1 and SpSub1 ssDBD dimer structures (40–105 and 40–94) were predicted using the Alphafold2 program [36] and superimposed to hPC4 (PDB 7E4W [35]) and MoSub1 (PDB 4AGH [9]) structures using the UCSF ChimeraX program [37]. The conserved residues are labeled in red, which are key for the interaction of the protein with the DNA and have been analyzed in our study. (**D**) Structural model of the complex formed by the ssDBD of ScSub1 (residues 32–105) and a single-stranded DNA molecule (dT10G) were obtained using the Hhpred interactive server. The two Sub1 monomers (ribbon in light and dark blue), the residues involved in DNA binding (sticks in red), and a DNA molecule (orange ribbon) are shown.

ScSub1 (292 amino acids) shows a strong similarity to human PC4 (127 amino acids) over a 65-residue region (amino acids 40–105), containing an ssDBD and sequences essential for co-activator function (Figure 1A; [19]). Budding yeast and fungus Sub1 ssDBD localize in the N-terminal region, whereas in the case of hPC4, it is located in the C-terminal region. Notably, in *S. pombe,* SpSub1 consists of 136 amino acids [8], and in *M. oryzae,* MoSub1 contains 162 amino acids [9], which is similar to PC4 in terms of size. However, *S. cerevisiae* Sub1 is significantly larger than its human, fission yeast, and fungus counterparts. Specifically, budding yeast Sub1 has an extra-long CT region of 187 amino acids [5,6,19], which is not conserved and is of unknown functionality. 

**Figure 2:**
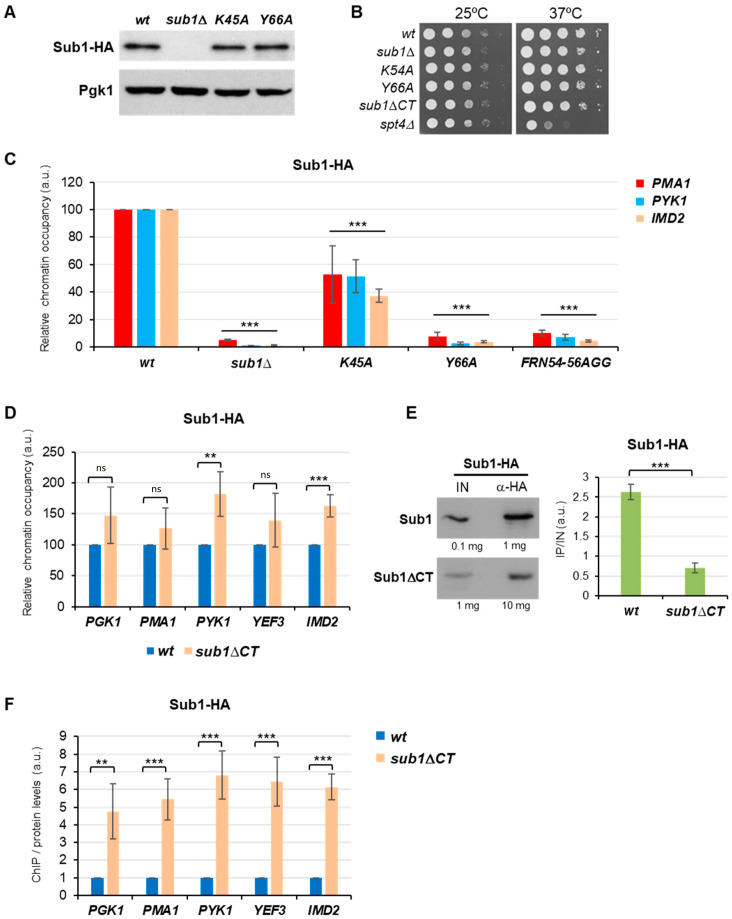
Evolutionarily conserved residues of ScSub1 ssDBD are crucial for its DNA binding capacity in vivo. (**A**) Analysis of Sub1-6HA levels in *wt* (Sub1-6HA), *sub1*Δ, and ssDBD mutants (*sub1-K45A*, *sub1-Y66A*) cells by Western blot. Levels of Pgk1 were used as a control of loaded proteins. (**B**) Yeast strains with the indicated genotypes were spotted onto solid media plates (1:10 serial dilutions) and grown at 28 °C or 37 °C for 2–3 days. *spt4*Δ cells were used as a control for cell growth and thermo-sensitivity defects. (**C**) ChIP analyses of Sub1-HA in the *wt*, *sub1*∆, and ssDBD mutants (*sub1-K45A*, *sub1-Y66A* and *sub1-FRN54-56AGG*). Sub1 occupancy at the 5′ region of two constitutively expressed genes, *PMA1* and *PYK1,* and the induced gene, *IMD2,* were examined by qPCR, and quantifications were graphed (see Materials and Methods). The numbers on the Y-axis represent the occupancy of Sub1 at the promoters in mutant cells relative to *wt* cells, in which occupancy is considered 100%. (**E**) ChIP analysis of *wt* and *sub1*∆*CT* cells expressing Sub1-6HA, as in (**D**). In this case, four constitutively transcribed genes were examined: *PGK1, PMA1, PYK1,* and *YEF3,* as well as *IMD2*. (**E**) Left: co-immunoprecipitation assays (Co IP) to estimate Sub1-HA levels in *wt* and *sub1*Δ*CT* cells; whole cell extracts (WCE) were prepared from strains expressing Sub1-6HA, and either 1 mg or 10 mg of WCE were used to immunoprecipitate Sub1 from *wt* or *sub1*Δ*CT* cells, respectively. Immunoprecipitated (α-HA) or inputs (IN)—0.1 mg and 1 mg, respectively—were loaded onto a 12% SDS-PAGE gel and immunoblotted with anti-HA. Right: chemiluminescence of immunoreactive bands from Western blots were quantified and graphed. (**F**) Ratio ChIP/protein levels with values obtained in D and E, respectively. In all cases, the mean and standard deviation values were calculated from at least three independent experiments. Statistically significant levels are shown where ns = non-significant, ** = *p* < 0.01, *** = *p* < 0.001.

We then aimed to investigate the contribution of the ssDBD and the CT region to Sub1′s functions in transcription. In the case of the ssDBD, Sub1 retains high sequence homology with PC4 and MoSub1 ssDBDs [9] (Figure 1A,B). It contains some residues that are considered key in the interaction of PC4 [13,15,17] and MoSub1 [7,9,16] with the DNA (Figure 1B,C). Neither ScSub1 nor SpSub1 ssDBD structures have been solved; thus, it is unknown if they dimerize, though it has been suggested [24]. Nevertheless, we modeled putative Sc and SpSub1 dimers and found that their predicted structures are quite similar to human and fungus orthologues when the structures were superimposed (Figure 1C). Indeed, the predicted model bound to the ssDNA (Figure 1D) shows that Sub1 would interact with the DNA in a manner very similar to that of hPC4 and MoSub1, reinforcing the hypothesis that some amino acids of the ssDBD are functionally conserved regarding the capacity to bind to DNA. Then, we decided to experimentally analyze the degree of the functional conservation of Sub1-ssDBD. As a first approach, we considered some of the residues present in PC4-ssDBD shown to be crucial for the in vitro interaction with the DNA and, then, mutated the corresponding residues in the Sub1 sequence. PC4 lysine 68 (K68), due to its positively charged side chain exposed to the solution and its position in the overall structure, forms hydrogen bonds to DNA and is important for the DNA binding capacity of PC4. Tryptophan 89 (W89) and phenylalanine 77 (F77) are particularly important, as they interact with various DNA residues through their aromatic side chains [13,14,15,17]. Aromatic residues are extremely conserved in their orthologs. Thus, tyrosine 57 (Y57) in SpSub1 and Y66, in ScSub1, localize in more equivalent positions than PC4-W89 [14,15] and MoSub1-Y74 [7,9] for their interactions with the DNA (Figure 1C). The mutation of W89 by alanine (W89A) severely affects the binding of PC4 to ssDNA in vitro [14], and similar effects display MoSub1-Y74 mutation [7]. Hence, this evolutionary conservation supports the crucial role of DNA interaction in PC4/Sub1 cellular functions [19], and the same may be true for ScSub1. Therefore, we investigated Sub1 ssDBD, generating two different mutants: *sub1-K45A*, in which K45 corresponds to PC4-K68, as well as *sub1-Y66A,* in which Y66 corresponds to PC4-W89 and MoSub1-Y74, which were replaced by alanine (Figure 1B). The DNA binding mutations were generated by site-directed mutagenesis of a wild-type copy of *SUB1* cloned into a centromeric plasmid under its own promoter. All the mutants and wild-type (*wt)* strains were tagged at the C-terminus of Sub1 with a 6xHA epitope, so protein levels were tested by Western blot. Levels of Sub1-K45A-HA and Sub1-Y66A-HA proteins were similar to those of *wt* Sub1-HA (Figure 2A).

To study the Sub1-CT region, we generated a mutant, deleting the CT region in the *SUB1* chromosomal copy (from K113 to the stop codon) and being replaced by 6xHA. We previously worked with an isogenic strain where a truncated copy of *SUB1*, containing only the ssDBD, was also cloned into a centromeric plasmid. We observed that Sub1ΔCT-HA protein levels were quite low, likely due to protein instability because we did not detect defect in transcription [29]. Similar effect is observed in Supplementary Figure S1A, where, in addition, the levels of the Sub1ΔCT-HA protein expressed from the chromosomal copy were analyzed but, in this case, were undetectable (Supplementary Figure S1). We needed to immunoprecipitate the protein or highly increase the amount of WCE in order to detect Sub1 without a functional CT region (Supplementary Figure S1A and Figure 2E). Apparently, in *sub1*Δ*CT* cells, Sub1 becomes very unstable, which suggests that the CT region may contain sequences important for Sub1′s stability.

We also analyzed the growth phenotypes of the *wt* and mutant cells, and we observed that noneof the mutants displayed growth defects at 28 °C, neither *SUB1* deletion (*sub1*Δ; Figure 2B). However, *sub1∆, sub1-Y66A*, and *sub1*∆*CT* strains show a very slight growth defect at 37 °C when compared with *wt* and *sub1-K45A* strains, in agreement with the fact that *SUB1* is a non-essential gene [25]. Next, in order to study Sub1 recruitment to gene promoters, we performed chromatin immunoprecipitation assays (ChIP) with the ssDBD mutants and *sub1*Δ*CT* cells. We already described that Sub1-HA association with chromatin was highest at the gene promoters and rapidly decreased in the coding regions of constitutively transcribed genes [26]. Further, we have also shown that, in *sub1-FRN54-56AGG* cells, Sub1-HA association was almost abolished [29], which agrees with the requirement of PC4-F77 for the interaction with the DNA (Figure 1B and [13,14,15]). Specifically, here, using ChIP, we have studied the effects of new mutations generated in the ssDBD of Sub1 (*sub1-K45A* and *sub1-Y66A*), including *sub1-FRN54-56AGG* cells, as well as positive and negative controls, Sub1-HA *wt* and *sub1*∆ cells, respectively. We then analyzed, in normal growth conditions, the association of Sub1-HA with *PMA1* and *PYK1* promoters and, in addition with *IMD2* promoter, whose expression depend on Sub1 [26]. In *wt* cells, Sub1-HA was efficiently recruited to the promoter of all of the analyzed genes (Figure 2C) but was dramatically reduced in the triple mutant, *sub1-FRN54-56AGG*, as we have already reported [29]. Similarly, Sub1 association was significantly decreased in *sub1-K45A* and almost nil in *sub1-Y66A* in the three studied genes, which agrees with the relevance of these conserved residues for PC4 DNA binding capacity and the strong homology between Sub1 and PC4 ssDBD (Figure 1, [13,14,15]). However, it has been reported that the Y66A mutation does not alter the binding of Sub1 to the *PYK1* promoter [24]. This is in contradiction with the crystal structure of the conserved ssDNA binding domain of PC4 in the context of a dimer, where W89 is essential for the interaction with the DNA [13,14,15], and with the fact that, in the Sub1 ssDBD dimer, predicted structure leads to a reliable model for Sub1 ssDNA binding domain (Figure 1C) in which Sub1-Y66 residue is located in the same position as PC4-W89 and MoSub1-Y74 when bound to ssDNA (Figure 1D). Next, we analyzed the contribution of the CT region of Sub1 to its ability to interact with gene promoters in *sub1*Δ*CT* cells. Interestingly, the association of Sub1ΔCT-HA with the chromatin of several genes was similar or slightly increased when compared to Sub1-HA in *wt* cells (Figure 2D). Nevertheless, in this case, Sub1ΔCT-HA protein levels are extremely low (Supplementary Figure S1A), and therefore, the observed effect is likely a consequence of reduced protein levels. To confirm this, we increased, by tenfold, the amount of immunoprecipitated (IP) Sub1-HA in *sub1*Δ*CT* to estimate the amount of the protein that associates with gene promoters in the mutant cells. However, there is still a higher level of Sub1 protein in *wt* than in *sub1*Δ*CT* (Figure 2E, left graph). In fact, when we calculated the ratio IP/input (IN), we observed that, in *wt* cells, approximately 3.75 times more Sub1 is immunoprecipitated (Figure 2E, right graph). We then normalized Sub1 occupancy values, at gene promoters obtained by qPCR (Figure 2D), to relative immunoprecipitated protein levels estimated by CoIP (Figure 2E). We observed that, in the absence of the CT region of Sub1, the relative association of Sub1 to gene promoters in the *sub1*Δ*CT* is around 4–6 times higher than in *wt* cells for the studied genes (Figure 2F). This result suggests that the CT region of Sub1 may not be required for Sub1 occupancy of gene promoters, but it can contribute to it, either by affecting the recruitment or release upon transcription initiation (see discussion). 

### 3.2. The C-Terminal Region of Sub1 Is Required for Proper Transcription Elongation

We have previously shown that Sub1 associates with coding regions in a transcription-dependent manner and influences transcription elongation rate [26]. According to the data shown in Figure 2, other than being part of the PIC, the CT region of Sub1 seems to be involved in other aspects of mRNA transcription and in the promotion of transcription initiation [24]. As deduced by the fact that deletion of Sub1-CT increases the relative occupancy of Sub1 to gene promoters (Figure 2G), even when protein levels are significantly reduced, we hypothesized that the CT of Sub1 could be required for Sub1 promoter release and/or to facilitate transcription elongation. 

To investigate if Sub1′s role in transcription elongation resides in the CT region, we used different strategies: (1) GLAM assays to study elongation efficiency; (2) reverse transcription, coupled with quantitative PCR (RT-qPCR), to analyze *IMD2* gene expression, which depends on a functional Sub1 [22,26]; (3) ChIP to evaluate RNAPII’s association with constitutive and regulated genes; (4) transcriptional run-on (TRO) assay to determine the levels of active elongation-competent polymerases.

**Figure 3:**
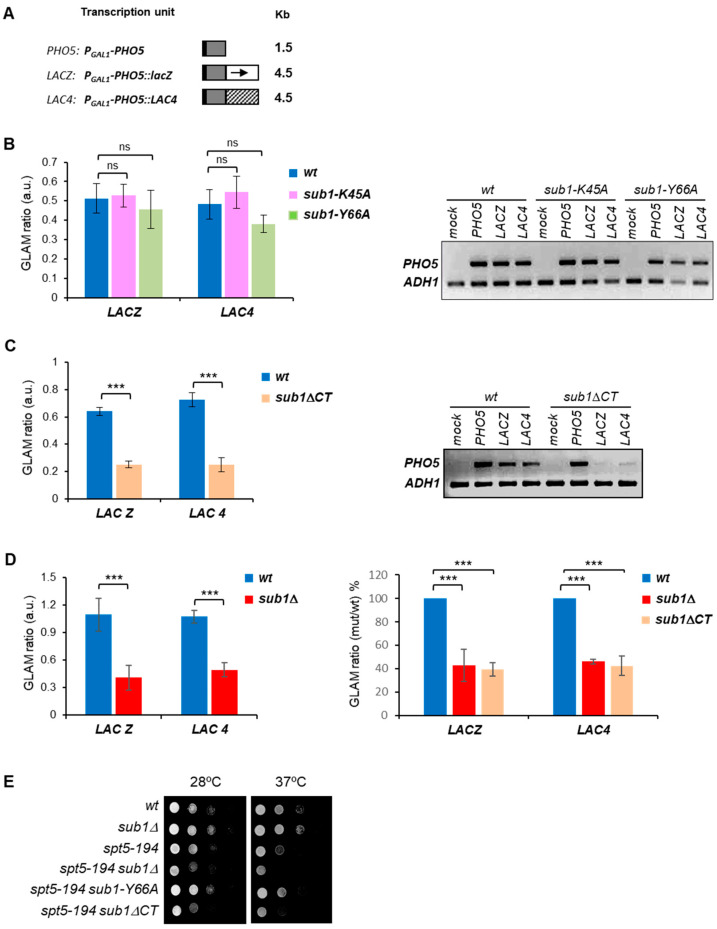
The deletion of the CT region of *SUB1* causes reduced transcription elongation efficiency of long transcripts. (**A**) A schematic representation of the three transcription units used in the GLAM assays. (**B**) The graph on the left shows the GLAM ratios, and the right panel shows *PHO5* expression from the short and long transcription units. As mock, a strain containing an empty plasmid was used. *ADH1* expression was used as a loading control. (**C**) GLAM ratios (left) and RT-PCR results (right) for the *wt* and *sub1*Δ*CT* strains. Statistically significant levels are shown where ns = non-significant, *** = *p* < 0.001. (**D**) Left, GLAM ratios for *wt* and *sub1*Δ. Right, comparison of transcription elongation defects between *sub1*Δ and *sub1*ΔCT relative to *wt*, set as 100%. (**E**) Allele-specific interaction between *SUB1*Δ*CT* and *SPT5*; the indicated yeast strains were spotted onto SC media plates and incubated at 28 °C or 37 °C for 2–3 days.

First, we measured the elongation efficiency by the GLAM assay (gene-length-dependent accumulation of mRNA), which specifically detects defects in elongation [34]. There were three plasmids containing the same *PHO5* (acid phosphatase) coding sequence under the control of the *GAL1* promoter, but with variable 3′ untranslated sequences, they were introduced into the wild-type (*wt*), ssDBD mutants (*sub1*-K45A and *sub1*-Y66A), and *sub1ΔCT* cells (Figure 3A). The GLAM ratios, defined as the acid phosphatase activity from the long transcripts versus to the short transcripts, were calculated for each strain (Figure 3B,C). As shown, the GLAM ratios for *sub1-K45A* and *sub1-Y66A* do not differ significantly from that of *wt* cells, whereas the ratios were dramatically reduced in the *sub1*Δ*CT* cells (Figure 3C, left panel), as well as in *sub1*Δ (Figure 3D, left panel), as we already reported [26]. In fact, the magnitude of the effect is quite similar for both mutants (*sub1*Δ and *sub1*Δ*CT*), when compared to *wt* cells (Figure 3D, right panel), and significantly lower than *wt*. Moreover, if we increase the amount of Sub1ΔCT-HA protein, we still observe a similar effect on transcription elongation (Supplementary Figure S1A,C). The gene-length-dependent defect observed in the *sub1*Δ*CT* cells was further validated by RT-PCR assays using the cDNA generated from all the strains expressing the different transcription units or containing an empty plasmid as a mock control (Figure 3B,C, right panels). Our results showed that *PHO5* expression is reduced in *sub1*Δ*CT* cells when expressed either as *PHO5*-lacZ or *PHO5*-*LAC4* long transcripts; however, the expression was not affected when *PHO5* was transcribed as a short transcript. In the case of ssDBD mutants, transcription of the *PHO5* transcripts was unaffected. Our data clearly indicate that, in the CT region of Sub1, the capacity of Sub1 to promote transcription elongation efficiency resides.

In a previous study, we demonstrated that Sub1 is genetically and functionally linked to Spt5 [26], which, together with Spt4, forms the evolutionarily conserved Spt4/5 complex essential for efficient transcription elongation by RNAPII [38,39,40,41]. To corroborate the role of the CT region of Sub1 in elongation, independent of the ssDBD, we conducted a genetic study taking advantage of the *spt5-194* mutation, which impairs RNAPII transcription elongation and grows slowly at 37 °C [39,42,43]. We then generated double mutant cells, combining the *spt5-194* mutation either with *sub1-Y66A* ssDBD mutation or with *sub1*Δ*CT*, and tested their growth at 28 °C and 37 °C. As already reported, *sub1*Δ cells grow normally, while *spt5-194* grows slowly at both temperatures, and *spt5-194 sub1*Δ double mutant shows increased sensitivity to temperature, when grown at 28 °C and 37 °C, as an indication of a negative genetic interaction (Figure 3D, [26]). Interestingly, no genetic interaction was observed between *spt5-194* and *sub1-Y66A*, but a clear genetic interaction was observed between *spt5-194* and *sub1*Δ*CT* because the *spt5-194 sub1*Δ*CT* double mutant grows worse than any of the single mutants. Indeed, these double mutants display growth defects similar to the double mutant *spt5-194 sub1*Δ that lacks *SUB1*. This allele-specific interaction reinforces the role of the CT region in transcription elongation, which seems to work independently of the ssDBD. Altogether, GLAM assay and genetic data show that, while the ssDBD is not required for transcription elongation, the lack of the CT region significantly impaired this process, indicating that in this region resides Sub1′s role in elongation.

**Figure 4:**
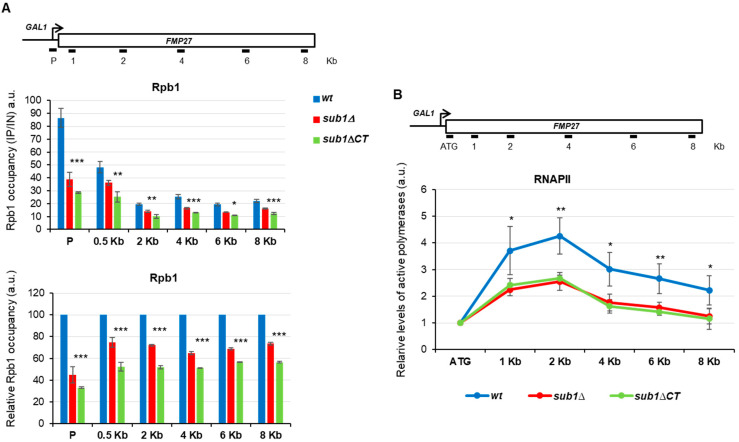
Decreased association of Rpb1 with chromatin and active elongation-competent RNAPII in *sub1*Δ*CT* cells during active transcription. (**A**) The upper panel is a schematic representation of the *GAL1-FMP27* gene; the PCR primer pairs, corresponding to the promoter and coding regions at the specified positions used in the ChIP assays, are indicated. The middle panel depicts the ChIP analysis of Rpb1 association with the *FMP27* gene upon galactose-induction in *wt*, *sub1*Δ, and *sub1*Δ*CT* cells; qPCR quantifications are shown in the graph, where the numbers on the Y-axis represent the ratio of the values obtained from specific primer products to the negative control (intergenic region of chromosome VII) and are normalized to the input controls. The bottom panel shows the relative Rpb1 occupancy within *FMP27*; qPCR values obtained for each analyzed gene region in mutant cells, *sub1*Δ and *sub1*Δ*CT*, were normalized to *wt* values, which were set to 100. (**B**) Transcriptional run-on (TRO); the upper panel is a diagram of the *GAL1-FMP27* gene, where locations of the probes used in the TRO assay are indicated. The lower panel shows the levels of active competent RNAPII from the TRO assay in *wt*, *sub1*Δ, and *sub1*Δ*CT* cells growing in a galactose-containing medium. After normalization for the 18S rRNA signal, the results were normalized to the ATG probe, which was fixed at 1, and plotted. Error bars represent standard deviations. Statistically significant levels are shown (ns = non-significant, * = *p* < 0.05, ** = *p* < 0.01, *** = *p* < 0.001).

To further corroborate the role of the Sub1-CT region in elongation, more specific and sophisticated methods were followed. Thus, to determine defects in elongation, we analyzed RNAPII association with chromatin, within gene bodies, during the transcription cycle by ChIP and measured active RNAPII engaged in transcription by transcriptional run-on (TRO). First, we investigated whether Sub1-CT plays a role in transcription elongation by testing Rpb1 (the largest subunit of RNAPII) occupancy from the promoter to 3′-end regions of the extra-long gene *FMP27* (7887 bp) during active transcription, taking benefit of a strain expressing a chromosomal construction where the gene is expressed under the control of the *GAL1* promoter. We measured the level of Rpb1 association with various positions within the 8Kb of the *GAL1-FMP27* fusion gene upon galactose induction in *wt*, *sub1*Δ, and *sub1*Δ*CT* cells (Figure 4A, top). As shown in the figure, RNAPII levels decreased from the promoter to the 3′-end region of the *FMP27* gene in *sub1*Δ cells, as already described [26], as well as in the *sub1*Δ*CT* mutant when compared with the *wt* cells (Figure 4A, middle panel). Indeed, when we normalized *sub1Δ* and *sub1*Δ*CT* qPCR values to those of *wt*, for each position within the *FMP27* gene (Figure 4A, bottom panel), we observed the decrease in Rpb1 association with chromatin all along the transcription unit in the mutant cells. The reduced association in the coding region could be a consequence of reduced Sub1 association during initiation or, in addition, due to a defect in transcription elongation. The ChIP assay measures the levels of RNAPII associated with chromatin during active transcription [44], though it cannot distinguish between active elongating polymerases and arrested or paused polymerases. However, the TRO assay detects nascent pre-mRNA, and it provides an estimation of the density of actively transcribing RNAPII [32,45]. We have already shown that Sub1 influences the level of active polymerases engaged in transcription elongation along the *GAL1-FMP27* [26].

Furthermore, we used the TRO assay to assess the distribution of transcriptionally competent polymerases distributed along the *FMP27* gene in *wt*, *sub1*Δ, and *sub1*Δ*CT* cells. We calculated the levels of active RNAPII along the gene, relative to the 5′ region (ATG; Figure 4B), to discard the effect observed in the promoter by the ChIP assay (Figure 4A); we observed a significant reduction in active polymerases in *sub1*Δ cells, as well as in the *sub1*Δ*CT* cells, compared with *wt* cells. This result indicates that the higher occupancy of Rpb1 along the *GAL1-FMP27* gene, detected by ChIP in the absence of full-length Sub1 or its CT regions (Figure 4A), is due to inactive, arrested polymerases that very likely cannot properly resume transcription. Taken together, the data presented here clearly sustain the role of the CT region of Sub1 in transcription elongation. 

### 3.3. Sub1 Participates in the Expression of the IMD2 Gene trough the CT Region

**Figure 5:**
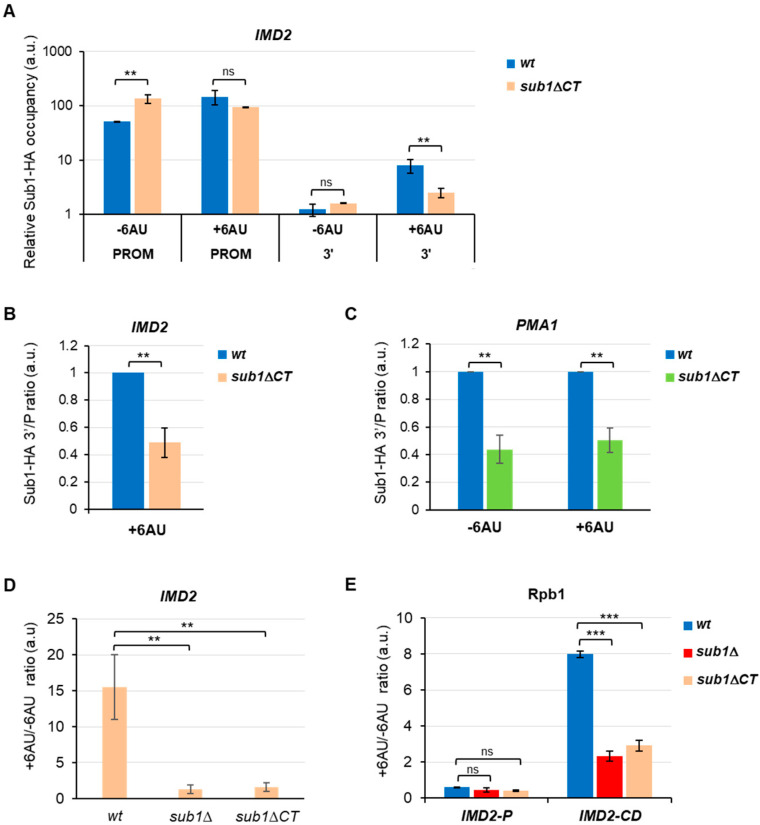
The C-terminal region of Sub1 is required for proper *IMD2* expression. (**A**) Sub1-HA relative occupancy at the promoter, as well as 3′ regions of the *IMD2* gene in *wt* and *sub1*Δ*CT* cells were analyzed by ChIP in the presence or absence of 100 μg/mL 6AU. Data represented in the graph were calculated and plotted as shown in Figure 4A; a logarithmic scale has been used to highlight the association defects at the 3′-end regions compared to the promoter region of the *IMD2* gene. (**B**) The 3′/P association ratio for *IMD2*. (**C**) The 3′/P association ratio for *PMA1* gene in the presence or absence of 100 μg/mL 6AU. (**D**) RT-qPCR to analyze *IMD2* gene expression; total RNA was purified from *wt*, *sub1*Δ, and *sub1*Δ*CT* cells grown in SC-URA, with or without 100 μg/mL 6AU, with synthesized cDNA and used qPCR reactions. The values from the reaction with *IMD2* mRNA were normalized to 18S rRNA, and the +6AU/-6AU ratio for *wt*, *sub1*Δ, and *sub1*Δ*CT* cells was calculated and graphed. (**E**) Relative Rpb1 occupancy, at the promoter and coding regions of the *IMD2* gene, was examined by ChIP in *wt*, *sub1*Δ, and *sub1*Δ*CT* cells before and after 6AU (100 μg/mL) treatment. The Rpb1 binding (promoter, P, and coding regions, CD) was measured in all the cells, as shown in (**A**), and then, the +6AU/-6AU ratio was estimated and represented. Error bars are standard deviations. Statistically significant levels are shown where ns = non-significant, ** = *p* < 0.01, ***= *p* < 0.001.

The drug 6-azauracil (6AU) is a potent inhibitor of inosine-5′-monophosphate (IMP) dehydrogenase, which catalyzes the rate-limiting reaction of *de novo* synthesis of GTP. In budding yeast, treatment of 6AU reduces cellular UTP and GTP levels [46], and cells respond by increasing the transcription of the *IMD2* gene, which encodes the IMP dehydrogenase [47,48]. *IMD2* induction is dependent upon a functional elongation machinery [48,49], and in consequence, mutations in the transcriptional elongation machinery exacerbate the cells’ sensitivity to 6AU. Thus, this drug has been widely used to sense transcription elongation defects (for instance, [39,49,50,51]). Remarkably, though Sub1 is an elongation factor, the *SUB1* deletion mutant is resistant to 6AU because the *IMD2* gene is constitutively expressed due to defects in the transcription start site selection. On the contrary, in isogenic *wt* cells, *IMD2* is induced only upon 6AU treatment [22,26,52]. Interestingly, we showed that Sub1 influences *IMD2* transcription elongation, thereby affecting the *novo* synthesis of *IMD2* after 6AU treatment [26]. To further investigate the function of the Sub1 CT region, we carried out ChIP experiments in *wt* and *sub1*Δ*CT* strains grown in the presence or absence of 6AU (Figure 5A; note that data are graphed on a logarithmic scale to compare Sub1 association with 5′ and 3′-ends). In *wt* cells, Sub1-HA binds to the *IMD2* promoter independently of 6AU treatment [26]. A similar association was detected in the *sub1*Δ*CT* mutant, though in contrast to *wt* cells, the Sub1ΔCT-HA association is slightly reduced after 6AU treatment. On the other hand, Sub1 was barely detected at the 3′ regions of the *IMD2* ORF in *wt* and *sub1*Δ*CT* cells in non-induction conditions, as expected. Upon exposure to 6AU, Sub1-HA binding in *wt* cells increased at the promoter and 3′ regions, which is in agreement with [26]; however, Sub1ΔCT-HA binding was very slightly reduced at the promoter and significantly reduced at the 3′-end of the *IMD2* gene (Figure 5A). In fact, when we calculated the 3′/P ratio for Sub1 binding after 6AU treatment (Figure 5B), we detected a dramatic reduction in the association of Sub1ΔCT-HA with the chromatin compared with *wt* Sub1-HA. Sub1ΔCT binds efficiently to the *IMD2* promoter region, so this reduction may be due to a defect in the release of Sub1 from the promoter, which thereby causes a decrease in Sub1 binding to the 3′ regions in *sub1ΔCT* cells following 6AU treatment, while it increases in *wt* cells. Similar results were obtained when we calculated the 3′/P crosslinking ratio for Sub1-HA and Sub1ΔCT-HA to the constitutive *PMA1* gene, in the presence or absence of 6AU (Figure 5C), as expected from a constitutive gene whose transcription also depends on Sub1 [26]. Accordingly, when we analyzed *IMD2* expression by RT-qPCR in *wt*, *sub1*Δ, and *sub1*Δ*CT* cells, we observed that the deletion of the C-terminal region behaved similarly to the *SUB1* deletion mutant (Figure 5D). *IMD2* was constitutively expressed in non-induction conditions, but its expression was reduced after 6AU treatment.

In summary, the *sub1*Δ*CT* mutant behaves similar to the *sub1*Δ mutant because the expression of *IMD2* cannot be induced during transcription elongation. In the case of the *sub1*Δ*CT* mutant, the function of Sub1 modulating the RNAPII rate during elongation would be affected [26], likely as a consequence of its stronger affinity for the chromatin in the promoter region, where most of Sub1 might be retained. In agreement, when we analyzed the association of Rpb1 with the *IMD2* gene, as a function of 6AU treatment (Figure 5E), we observed that the loss of the C-terminal domain of Sub1 provoked a decrease in Rpb1 crosslinking to coding regions, similar to *sub1*Δ. All these data indicate that Sub1-HA actively participates in *IMD2* transcription elongation via its CT region.

## 4. Discussion

### 4.1. PC4/Sub1 ssDNA Binding Domain Is Functionally Conserved

Several orthologues of the human positive coactivator PC4 have been discovered in bacteriophages, bacteria, and eukaryotes, showing that it is an evolutionarily conserved factor. All of them are involved in different aspects of DNA metabolism and are characterized by the presence of an ssDBD [7,8,9,10,11,12]. The structure of this domain, as a dimer bound to the DNA, has been solved in several of these organisms, disclosing an extraordinary similitude, for instance, between humans [13,14,15] and rice [7,9,16]. The hPC4 and MoSub1 proteins contain key conserved residues which are involved in the interaction with the DNA and are present in budding and fission yeasts’ Sub1 (Figure 1B). Indeed, structures predicted for ScSub1 and SpSub1 ssDBD dimers report reliable models (Figure 1C), suggesting that the interaction between the yeast’s orthologues proteins and the DNA is also highly conserved. The data presented here and in the literature [29] support the hypothesis of functional conservation of the ScSub1 ssDBD, likely also a structural conservation, because mutations of key residues in ScSub1 either significantly reduce its DNA binding capacity (K45A) or almost abolish it (Y66A and FRN54-56AGG) in vivo (Figure 1B and Figure 2D), as it has been shown in vitro for PC4 [14,15] and MoSub1 [7]. Notably, we have shown that altering ScSub1′s DNA binding capacity does not affect its role of transcribing long transcripts (Figure 3B), indicating that the ssDBD is not involved in transcription elongation, at least directly, and its principal role might be to recruit Sub1 to gene promoters (Figure 2D). Accordingly, PC4 ssDBD is involved in transcriptional activation [4,5,6], and similarly, Sub1 is required for the activation of osmo-response genes [21] and to respond to DNA damage [53]. Moreover, the ssDBD of PC4 and ScSub1 are also implicated in transcription repression [14,22]. To date, all the functions described for PC4/Sub1 ssDNA binding domains have been related to transcription initiation. Our data corroborate it and add further knowledge, unveiling key residues localized within this domain, which are essential for the DNA interaction, similar to hPC4, and therefore, demonstrating their evolutionarily functional conservation.

### 4.2. S. cerevisiae Sub1 Contains an Unusual CT Extension with a Role in Transcription Elongation

PC4/Sub1 proteins are similar in size, except for the budding yeast Sub1 (Figure 1A), which contains an extra-long C-terminal region, non-conserved in other organisms (ScSub1; Figure 1A). Although no roles have been assigned to this region up to date, it is considered indispensable for the roles of Sub1 associated to its DNA binding capacity; for instance, *IMD2* transcription repression and the response to DNA damage [22,53]. However, we have presented evidence here indicating that the C-terminal region of Sub1 might regulate its DNA binding capacity because the absence of this region induces an increase in the association of Sub1 with gene promoters, which results in its retention at this location and, consequently, in a defect in elongation, as determined by GLAM, ChIP, TRO, and RT-qPCR assays (Figure 2E–G, Figure 3, Figure 4 and Figure 5).

The truncation of the CT region produces a very low amount of the Sub1 protein, which is not due to defects in transcription, but it is likely due to protein instability/degradation. It is unknown if this region contains target sequences for Sub1 degradation. In any case, although low levels of the Sub1ΔCT protein only containing a functional ssDBD are produced, the interaction of the truncated protein with the gene promoters is stronger than in the case of a wild-type Sub1 protein (Figure 2F). Therefore, the CT region might be implicated in other functions, such as contributing to Sub1 binding to the DNA during initiation, as part of the PIC [24], and/or promoting its release upon initiation to join the elongation complex [26] through the interaction with transcription factors. In agreement with it, some years ago we found that the CT region of Sub1 is functionally and genetically linked to Rpb4/7, the stalk domain of RNAPII [29]. Indeed, our data indicated that Sub1 stays associated with the RNAPII during the whole transcription while interacting with Rpb4/7 [29]. In addition, we previously showed that Sub1 also associates with the elongation factor Spt5 to promote efficient transcription elongation [26]. Strongly supporting the role of the CT in elongation, we have found an allelic genetic interaction between *sub1*∆*CT* and *spt5-194*, which is not observed in the case of the ssDBD mutations (Figure 3D). Remarkably, Spt5 also targets the stalk and clamp domains of RNAPII, and both domains are functionally and genetically linked to Sub1 [20,29,54,55]. Altogether, our data suggest that the CT region of Sub1 could act as a switch between transcription initiation and elongation, becoming critical for transcription elongation efficiency.

### 4.3. Understanding the Mechanisms Underlying ScSub1 Functions

The phosphorylation of PC4 negatively influences its dsDNA binding capacity, thereby fostering its escape from promoters [4,56,57]. In this regard, very recently, it has been reported that mutations of SpSub1 S98 and S100 in the CT region of the protein, just after the ssDBD, disrupt Sub1 phosphorylation in vitro by CK2, and the phosphorylation of these two residues negatively influences in vitro transcription, as in the case of PC4 [58]. However, in the human protein, the phosphorylated residues are in a serine-rich region, SEAC (Figure 1A; [4,56,57]). The authors of this study argue that, although the location of the phosphorylated residues in fission yeast is the C-terminus, the functional consequence of PC4/SpSub1 phosphorylation is the same, which is the inhibition of in vitro basal transcription [58]. It has been previously reported that ScSub1 can also be phosphorylated in vitro and that phosphorylated recombinant Sub1 binds weaker to DNA than the unphosphorylated protein [5]. Although it is unknown whether Sub1 binding capacity is regulated in vivo by phosphorylation, our results indicate that the extra C-terminal region, directly or indirectly, impacts Sub1 interaction with DNA. We cannot discard the possibility that the CT region could be phosphorylated, as occurs in PC4 [4,56,57] or SpSub1 [4,56,57], to facilitate Sub1’s exit from the promoter and, then, regulate transcription and influence pre-mRNA processing (splicing and polyadenilation) [19,20,25,26,27,28,29]. We used the AlphaFold Protein Structure Database to predict Sub1 full length protein structure (Figure S2), and we localized all the phospho-sites identified in several phospho-proteomic studies (S119, Y123, S160, S263, S268, S269, S276, and S289) [59,60,61,62,63,64,65,66,67]. Curiously, all phospho-residues are within the CT region: two of them are near the ssDBD, and five of them are in the most C-terminal region of the protein. Phosphorylation of all or some of these residues upon transcription initiation could affect the DNA binding capacity of it and/or allow it to interact with other components of the transcription machinery, such as Spt5, to participate in the elongation process [26,29,68]. Indeed, Spt5 needs to be phosphorylated by the kinase Bur1, whose activity is modulated by Sub1, to stimulate transcription elongation [69,70]. This occurs early during the initiation/elongation transition, where Sub1 joins the elongation complex [26]. The confirmation that Sub1 is subject to these post-translational modifications (PTMs) or others in vivo would help to understand how the function of Sub1 is regulated: for instance, whether Sub1 phosphorylation modulates the DNA binding capacity or its release from the promoters upon initiation to facilitate efficient transcription elongation. On the other hand, dephosphorylation of Sub1 would help to dissociate it from the transcription complex upon transcription termination, as we proposed [27] and as is the case for other factors, including RNAPII [71]. Consistent with it, Sub1 has been genetically and functionally linked to RNAPII kinases and phosphatases [25,29,68]. Sub1 PTMs and, in particular, phosphorylation will help to understand the mechanism by which it influences all the stages of transcription [19,20].

Overall, our data indicate that the C-terminal region of Sub1 is key to understanding the dual role of Sub1 in transcription, which possibly occurs via the promotion of transition from initiation to elongation. Further, the data corroborate that Sub1 can act as a general regulator of RNAPII transcription during the whole transcription cycle by using different protein regions. Future research must precisely dissect the Sub1CT region and determine whether it contains distinct and functional domains, including sequences involved in its stability, and if its phosphorylation regulates Sub1 functions.

## Data Availability

Not applicable.

## Supplementary Materials

The following supporting information can be downloaded at: https://www.mdpi.com/article/10.3390/cells11203320/s1, Table S1: Yeast strains, Figure S1: The deletion of Sub1-CT severely affects Sub1 protein levels, Figure S2: Phosphorylation of Sub1-CT could be important for Sub1 function regulation. References [72,73] are cited in the Supplementary Materials.
