# Supplementary material for: The Role of S. cerevisiae Sub1/PC4 in Transcription Elongation Depends on the C-Terminal Region and Is Independent of the ssDNA Binding Domain
Source: Cells. 2022 Oct 21;11(20):3320. doi: 10.3390/cells11203320 (PMC9600219; doi:10.3390/cells11203320)
Supplement: Supplementary file 1 [file cells-11-03320-s001.zip › cells-1930854-supplementary.pdf]

SUPPLEMENTARY MATERIALS

Table S1. Yeast strains

Strain	Genotype	Source
OCSC1601	*mat a, his3Δ1 leu2ΔO met15Δ0 ura3ΔO pRS316 [URA3 CEN]*	This study
OCSC1602	*mat a, his3Δ1 leu2ΔO met15Δ0 ura3ΔO sub1::HIS3 [URA3 CEN]*	This study
OCSC1605	*mat a, his3Δ1 leu2ΔO met15Δ0 ura3ΔO sub1ΔCT::HIS3 [URA3, TRP1 CEN]*	This study
MGSC339	*mat α ade2-1 can1-100 his3-11,15 leu2,3 trp1-1 ura3-1 spt4::URA3*	A. Aguilera
OCSC1739	*mat a ade2-1 his 3-11,15 leu2-3,112 trp1-1 ura3-1 sub1::KAN [URA3 CEN]*	This study
OCSC1740	*mat a ade2-1 his 3-11,15 leu2-3,112 trp1-1 ura3-1 sub1::KAN [SUB1-6HA::TRP1 HIS3 CEN]*	This study
OCSC1712	*mat a ade2-1 his 3-11,15 leu2-3,112 trp1-1 ura3-1 sub1::KAN [SUB1-ΔCT-6HA::TRP1 HIS3 CEN]*	[2]
OCSC1742	*mat a ade2-1 his 3-11,15 leu2-3,112 trp1-1 ura3-1 sub1::URA3 [SUB1-FRN54-56AGG-6HA::TRP1 CEN]*	This study
OCSC1743	*mat a ade2-1 his 3-11,15 leu2-3,112 trp1-1 ura3-1 sub1::URA3 [SUB1-K45A-6HA::TRP1 CEN]*	This study
OCSC1744	*mat a ade2-1 his 3-11,15 leu2-3,112 trp1-1 ura3-1 sub1::URA3 [SUB1-Y66A-6HA::TRP1 CEN]*	This study
OCSC1434	*mat a ade2-1 his 3-11,15 leu2-3,112 trp1-1 ura3-1 Sub1-6HA::TRP1*	This study
OCSC2055	*mat a ade2-1 his 3-11,15 leu2-3,112 trp1-1 ura3-1 SUB1-ΔCT-6HA::TRP1*	[2]
GHY611	mat a his4-912δ leu2Δ1 trp1Δ63 *lys-128δ SPT5-MYC*	[3]
GHY94	*mat a his3Δ200 leu2Δ1 trp1Δ63 ura3-52 lys-128δ spt5-194*	G. Hartzog
OCSC1658	*mat a his3Δ200 leu2Δ1 trp1Δ63 ura3-52 lys-128δ spt5-194 [URA3, HIS3 CEN]*	This study
OCSC181	*mat a his3Δ200 leu2Δ1 trp1Δ63 ura3-52 lys-128δ spt5-194 sub1::URA3 [HIS3 CEN]*	[1]
OCSC1660	*mat a his3Δ200 leu2Δ1 trp1Δ63 ura3-52 lys-128δ spt5-194 sub1::URA3 [SUB1-6HA::TRP1 HIS3 CEN]*	This study
OCSC1663	*mat a his3Δ200 leu2Δ1 trp1Δ63 ura3-52 lys-128δ spt5-194 sub1::URA3 [SUB1-Y66A-6HA::TRP1 HIS3 CEN]*	This study
OCSC1661	*mat a his3Δ200 leu2Δ1 trp1Δ63 ura3-52 lys-128δ spt5-194 sub1::URA3 [SUB1-ΔCT-6HA::TRP1 HIS3 CEN]*	This study
MGSC339	*mat α ade2-1 can1-100 his3-11,15 leu2,3 trp1-1 ura3-1 spt4::URA3*	[1]
GYLR-3A	*mat a, his3Δ1 leu2ΔO met15Δ0 ura3ΔO TRP1::HISG URA3::GAL1-YLR454w (FMP27)*	[4]
OCSC1768	*mat a, his3Δ1 leu2ΔO met15Δ0 ura3ΔO TRP1::HISG URA3::GAL1-YLR454w sub1::KAN*	[1]
OCSC1769	*mat a, his3Δ1 leu2ΔO met15Δ0 ura3ΔO TRP1::HISG URA3::GAL1-YLR454w sub1ΔCT::KAN*	This study

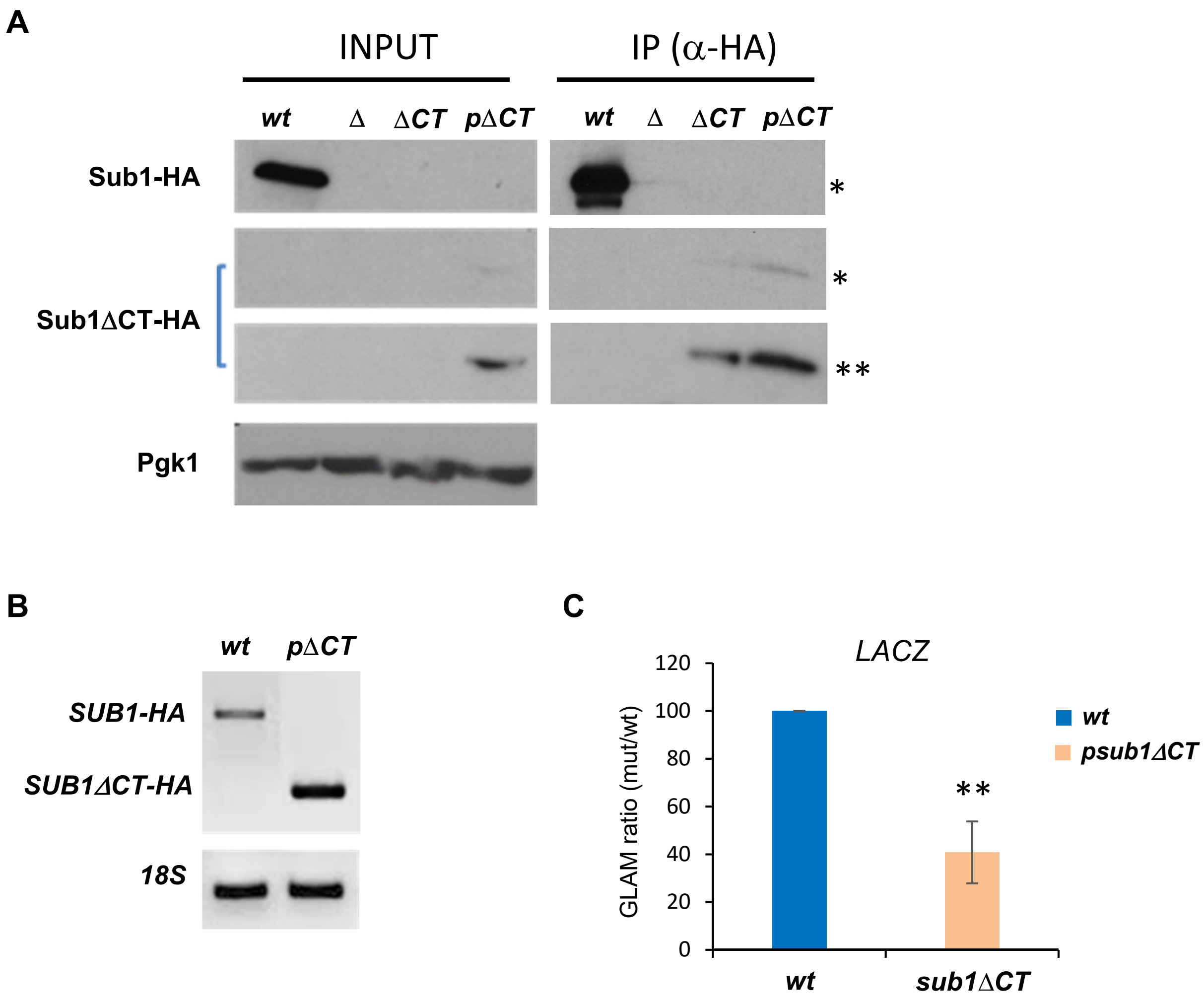

Figure S1. The deletion of Sub1-CT severely affects Sub1 protein levels. (A) Sub1-HA CoIP. Left panel: analysis of Sub1-6HA levels in *wt* and *sub1ΔCT-6HA*, expressed either from the chromosomal copy (ΔCT) or from a centromeric plasmid (pΔCT) in whole cell extracts (INPUT); *sub1Δ* cells were used as negative control and levels of Pgk1 as a loading control. Right panel: Immunoprecipitation of Sub1-HA (**) using anti-HA antibody and WCE from the indicated strains. Two exposure of the same blot are shown: (*) indicates same time of exposition, and (**) it is and over-exposure to detect Sub1ΔCT protein. (B). Analysis of *SUB1-HA* expression by RT-PCR in *wt* and *sub1ΔCT* cells, expressing *SUB1* from a plasmid, and using primers annealing at the 5′ region of *SUB1* and downstream at the HA epitope. 18S rRNA expression was used as a control. (C) **Increasing Sub1ΔCT protein levels does not improve transcription elongation efficiency.** GLAM ratio of the *wt* strain, and the *sub1Δ* strain expressing several copies of Sub1ΔCT-HA protein from a centromeric plasmid (p*Δ*CT in (A). Relative values of acid phosphatase activity from the *PHO5-LACZ* long transcript are shown, where *wt* values has been set as 100. The GLAM ratio for the *sub1ΔCT* strain is significantly lower than for *wt* cells. Significant level ** = $p < 0.01$.

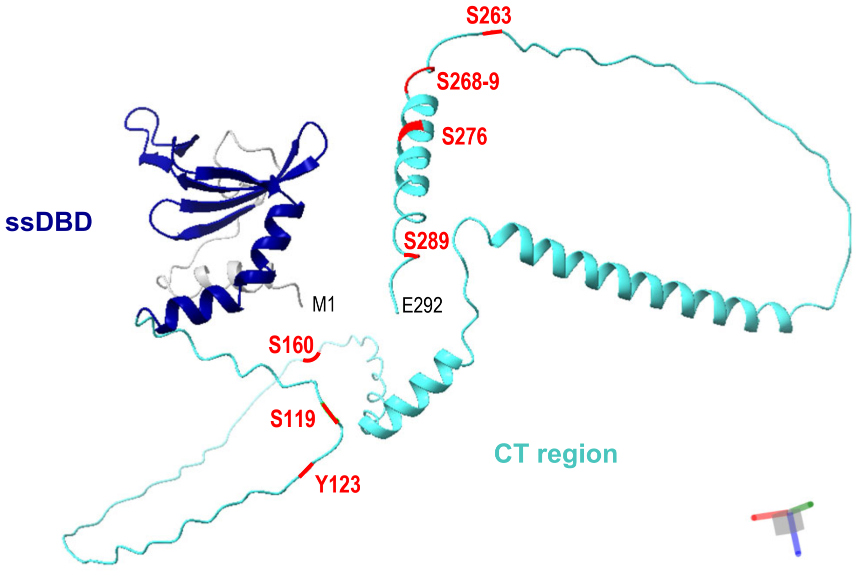

Figure S2. Phosphorylation of Sub1-CT could be important for Sub1 function regulation. ScSub1 structure as predicted by the AphaFold2 program [36] (https://alphafold.ebi.ac.uk/entry/P54000). The ssDBD is shown in dark blue, and the CT region in light blue. Phosphorylation sites described in proteomic studies [59–67] are indicated in red.

References

1. Garcia, A.; Collin, A.; Calvo, O. Sub1 associates with Spt5 and influences RNA polymerase II transcription elongation rate. *Mol Biol Cell* **2012**, *23*, 4297-4312, doi:10.1091/mbc.E12-04-0331.
2. Garavis, M.; Gonzalez-Polo, N.; Allepuz-Fuster, P.; Louro, J.A.; Fernandez-Tornero, C.; Calvo, O. Sub1 contacts the RNA polymerase II stalk to modulate mRNA synthesis. *Nucleic Acids Res* **2017**, *45*, 2458-2471, doi:10.1093/nar/gkw1206.
3. Lindstrom, D.L.; Squazzo, S.L.; Muster, N.; Burckin, T.A.; Wachter, K.C.; Emigh, C.A.; McCleery, J.A.; Yates, J.R., 3rd; Hartzog, G.A. Dual roles for Spt5 in pre-mRNA processing and transcription elongation revealed by identification of Spt5-associated proteins. *Mol Cell Biol* **2003**, *23*, 1368-1378.
4. Jimeno-Gonzalez, S.; Haaning, L.L.; Malagon, F.; Jensen, T.H. The yeast 5'-3' exonuclease Rat1p functions during transcription elongation by RNA polymerase II. *Mol Cell* **2010**, *37*, 580-587, doi: 10.1016/j.molcel.2010.01.019.
